# Effects of Anethole in Nociception Experimental Models

**DOI:** 10.1155/2014/345829

**Published:** 2014-11-25

**Authors:** Alessandra Mileni Versuti Ritter, Franciele Queiroz Ames, Fernando Otani, Rubia Maria Weffort de Oliveira, Roberto Kenji Nakamura Cuman, Ciomar Aparecida Bersani-Amado

**Affiliations:** ^1^Department of Pharmacology and Therapeutics, Laboratory of Inflammation, State University of Maringá, Avenida Colombo 5790, 87020-900 Maringá, PR, Brazil; ^2^Department of Pharmacology and Therapeutics, Laboratory of Brain Ischemia and Neuroprotection, State University of Maringá, Maringá, PR, Brazil

## Abstract

This study investigated the antinociceptive activity of anethole (anethole 1-methoxy-4-benzene (1-propenyl)), major compound of the essential oil of star anise (*Illicium verum*), in different experimental models of nociception. The animals were pretreated with anethole (62.5, 125, 250, and 500 mg/kg) one hour before the experiments. To eliminate a possible sedative effect of anethole, the open field test was conducted. Anethole (62.5, 125, 250, and 500 mg/kg) showed an antinociceptive effect in the writhing model induced by acetic acid, in the second phase of the formalin test (125 and 250 mg/kg) in the test of glutamate (62.5, 125, and 250 mg/kg), and expresses pain induced by ACF (250 mg/kg). In contrast, anethole was not able to increase the latency time on the hot plate and decrease the number of flinches during the initial phase of the formalin test in any of the doses tested. It was also demonstrated that anethole has no association with sedative effects. Therefore, these data showed that anethole, at all used doses, has no sedative effect and has an antinociceptive effect. This effect may be due to a decrease in the production/release of inflammatory mediators.

## 1. Introduction

Anethole (1-methoxy-4-benzene-[1-propenyl]) is a phenylpropanoid that is principally obtained from the essential oil of star anise (*Illicium verum*), anise (*Pimpinella anisum*), and sweet anise (*Foeniculum vulgare*) and comprises more than 90% of the essential oil of these plants [1, 2]. Anethole is found in lower concentrations in the essential oils of lemon balm (6.1%), cilantro (0.5%), and basil (0.08%) [3].

Anethole appears to be responsible for most of the properties attributed to the essential oil of star anise, with antioxidant [4], anticarcinogenic [5, 6], anti-inflammatory [3, 7], and antihypernociceptive [8] actions, among others. Some studies have demonstrated that the antioxidant effect of anethole is attributable to its ability to sequester free radicals, thus increasing the intracellular levels of glutathione and glutathione-*S*-transferase and inhibiting lipid peroxidation [3]. The ability to suppress the incidence and development of carcinomas is not yet well understood [3, 9]. Choo et al. [6] recently demonstrated that its anticarcinogenic activity is related to inhibitory effects on cellular adhesion and tumor invasion and the suppression of proteolytic enzymes through the nuclear factor *κ*B (NF-*κ*B) signaling pathway.

Recently we showed that oral anethole administration in experimental animals inhibited the formation of inflammatory exudate and migration of leukocytes in models of pleurisy induced by carrageenan and ear edema induced by croton oil, antihypernociceptive activity in models of acute and persistent inflammatory pain induced by carrageenan and complete Freund adjuvant (CFA), respectively. We also demonstrated that anethole reduces the recruitment of neutrophils in experimental models of* in vitro* chemotaxis and* in situ* microcirculation [10], in addition to immunomodulatory activity through a reduction of the migration of lymphocytes and macrophages induced by sheep erythrocytes antigens (unpublished date).

Some of these effects have been related to the inhibitory effect of anethole on the production or release of inflammatory mediators, such as prostaglandins, nitric oxide [7], interleukin-1 (IL-1), tumor necrosis factor (TNF), and IL-17 [8]. Additionally, we demonstrated that anethole did not alter the plasma levels of transaminases (aspartate transaminase and alanine transaminase, i.e., markers of hepatic lesions) or morphological and histological profiles of hepatic tissue when administered for 7 days [8].

Despite these findings, there was a need to continue the studies on the antinociceptive activity of anethole. Therefore, our aims in this study were to investigate the role of anethole in various experimental models of nociception. The mechanisms involved in this activity have been discussed.

## 2. Materials and Methods

### 2.1. Animals

The experiments were performed in male Swiss mice, weighing 20–30 g. They were obtained from the breeding stock of the Laboratory of Inflammation, University of Maringá, PR, Brazil. The mice were housed in a temperature-controlled room on a 12 h/12 h light/dark cycle with* ad libitum* access to standard rat chow and water. The experimental protocols were approved by the Committee for Animal Studies of the State University of Maringá (125/2010, CEEA).

### 2.2. Protocol of Treatment

The animals were treated orally with anethole (62.5, 125, 250, and 500 mg/kg), the reference antiinflammatory drug indomethacin (10 mg/kg), or saline (10 mL/kg) as a negative control. The schedules of treatment were determined on the basis of previous literature [7, 8]. The drugs were prepared immediately prior to use.

### 2.3. Acetic Acid-Induced Writhing

The mice were treated with anethole (62.5, 125, 250, and 500 mg/kg, p.o.), indomethacin (10 mg/kg, p.o.), or saline (10 mL/kg, p.o.). One hour after treatment, acetic acid solution (0.6%) was injected into the peritoneal cavity. The mice were then placed in a larger glass cylinder, and the intensity of nociceptive behavior was quantified by counting the total number of writhes that occurred 0–20 min after the injection.

### 2.4. Formalin Test

The formalin-induced paw kicking test was performed as described by Hunskaar and Hole [11], with some modifications. The mice were pretreated with different doses of anethole (62.5, 125, 250, and 500 mg/kg, p.o.), indomethacin (10 mg/kg, p.o.), or saline (10 mL/kg, p.o.). One hour after treatment, 20 *μ*L of a 2.0% formalin solution was injected into the plantar surface of the hind paw. The animals were then individually placed in glass cylinders. Nociceptive behavior was determined by the number of flinches induced by formalin. Kicking of the injected paw was counted from 0 to 5 min (first phase) and from 15 to 40 min (second phase) after the formalin injection. These phases correspond to neurogenic and inflammatory pain responses, respectively.

### 2.5. Complete Freund Adjuvant-Induced Pain

The animals were pretreated with anethole (250 mg/kg, p.o.) or saline (10 mL/kg, p.o.). One hour later, 20 *μ*L of complete Freund adjuvant (CFA) was injected into the plantar surface of the hind paw. The animals were observed from 0 to 20 min, and the time they spent licking the injected paw was recorded and considered indicative of nociception.

### 2.6. Hot-Plate Test

The animals were placed on a 55.0 ± 0.5°C hot plate (Ugo Basile Varese, Italy). Reaction times were recorded when the animals licked or kicked the hind paw or jumped 15, 30, 60, and 90 min after administration of anethole (125, 250, and 500 mg/kg, p.o.), saline (10 mL/kg, p.o.), or the reference drug meperidine (50 mg/kg, i.p.). The baseline was the mean reaction time of each animal, and a cutoff of 30 s was used to avoid tissue damage.

### 2.7. Glutamate Test

The glutamate test was performed according to Beirith et al. 2002 [12], with some modifications. The animals were pretreated with anethole (62.5, 125, and 250 mg/kg, p.o.). One hour later, 20 *μ*L of a glutamate solution (10 *μ*mol/paw) was injected under the ventral surface of the left hind paw. After the intraplantar injection of glutamate, the animals were individually placed into glass cylinders (20 cm diameter), and the time spent licking and biting the injected paw was recorded with a chronometer. This time was considered indicative of pain. The mice were observed for 15 min. Paw edema was measured 15 min after glutamate injection using plethysmometry.

### 2.8. Open-Field Test

Locomotor activity was quantified for 5 min in an open field that consisted of a white Plexiglas box (45 × 45 cm) without physical barriers. One hour after anethole treatment (62.5, 125, 250, and 500 mg/kg, p.o.) and 7 days after anethole treatment (250 mg/kg), each mouse was gently placed in the center of the box, and locomotor activity was scored. Behavior was continuously recorded by a video camera that was placed over the apparatus and encoded using ANY-maze software.

### 2.9. Statistical Analysis

The statistical analysis was performed using Prism software (GraphPad, San Diego, CA, USA) and analysis of variance (ANOVA). Values of *P* < 0.05 were considered statistically significant.

## 3. Results

### 3.1. Acetic Acid-Induced Writhing

**Figure 1:**
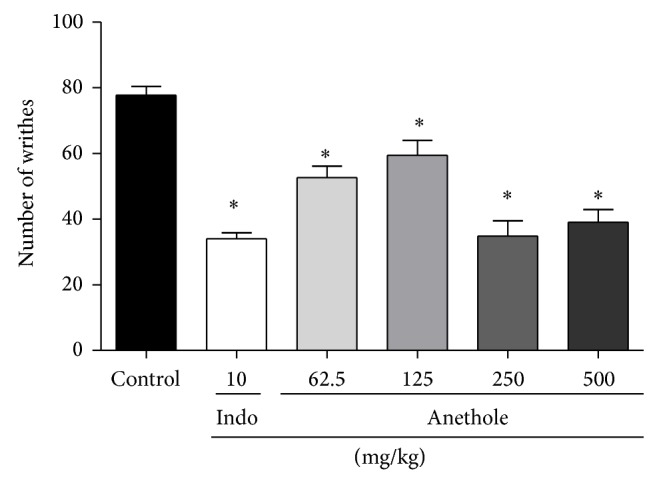
Effect of oral administration of anethole on acetic acid-induced writhing in mice. 0.6% acetic acid was intraperitoneally injected to mice 60 min after administration of the anethole (62.5, 125, 250, and 500 mg/kg, p.o.) and indomethacin (10 mg/kg, p.o.). Data are represented as mean ± S.E.M. ^*^ *P* < 0.05, compared with the control group (one-way ANOVA followed by Tukey test).

After the acetic acid solution injection, the mice exhibited 7.7 ± 2.6 abdominal constriction in the control group. Treatment with different doses of anethole or indomethacin significantly suppressed abdominal constrictions compared with controls. The inhibition percentages were 56% for indomethacin and 32%, 24%, 55%, and 50% for anethole at doses of 62.5, 125, 250, and 500 mg/kg, respectively (Figure 1).

### 3.2. Formalin Test

**Figure 2:**
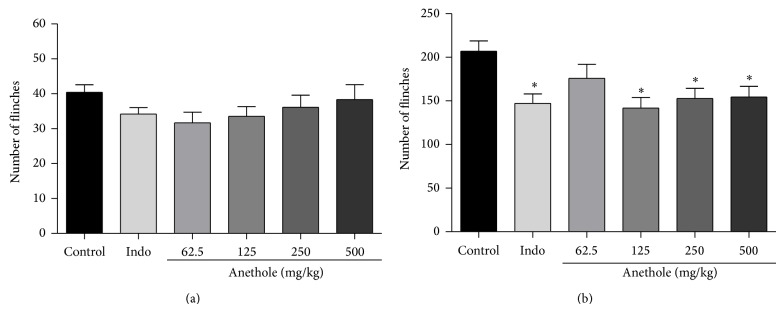
The effects of anethole on the early phase (a) (0–5 min) and late phase (b) (15–40 min) on 2% formalin-induced inflammation in mice. 20 *μ*L of 2% formalin solution was injected into the dorsal surface of the hind paw of mice 60 min after administration of the anethole (62.5, 125, 250, and 500 mg/kg, p.o.) and indomethacin (10 mg/kg, p.o.). Data are represented as mean ± S.E.M. ^*^ *P* < 0.05, compared with the control group (one-way ANOVA followed by Tukey test).

Treatment with indomethacin and the different doses of anethole did not reduce the number of kicks (flinches) compared with the control group in the first phase of the formalin test (Figure 2(a)), which assesses neuropathic pain. However, indomethacin and anethole at doses of 125 and 250 mg/kg significantly reduced the number of kicks (flinches) induced by formalin compared with control group in the second phase of the test, which assesses inflammatory pain. The inhibition percentages were 29% for indomethacin and 31%, 26%, and 25% for anethole at doses of 125, 250, and 500 mg/kg, respectively (Figure 2(b)).

### 3.3. Hot-Plate Test

The animals in the control group remained on the hot plate for an average of 8.4 ± 0.5 seconds. Meperidine (50 mg/kg) significantly increased the response latency compared with the control group (25.4 ± 1.7, 14.0 ± 1.3, 14.2 ± 1.2 seconds) 15, 30, and 60 min after treatment, respectively. However, anethole at different doses did not significantly increase the response latency on the hot plate at any of the periods analyzed (Table 1).

### 3.4. Glutamate Test

**Figure 3:**
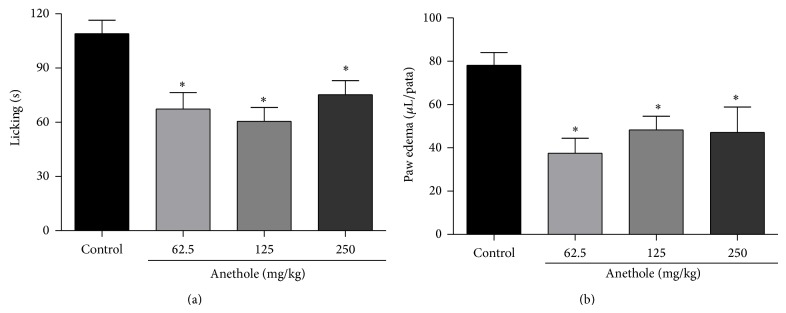
Effect of oral treatment of anethole (62.5, 125, and 250 mg/kg) on time spent licking (a) and paw edema (b) after intraplantar injection of glutamate (10 *μ*mol/paw) in mice. Data are represented as mean ± S.E.M. ^*^ *P* < 0.05, compared with the control group (one-way ANOVA followed by Tukey test).

The glutamate injection induced a nociceptive response, reflected by the time the animals spent licking the paw and paw edema formation. Anethole at doses of 62.5, 125, and 250 mg/kg exerted significant antinociceptive activity compared with the control group. The inhibition percentages were 38%, 44%, and 31% for the 62.5, 125, and 250 mg/kg doses, respectively (Figure 3(a)). Treatment with anethole significant reduced paw edema compared with the control group, with inhibition percentages of 52%, 38%, and 40% for the doses of 62.5, 125, and 250 mg/kg, respectively (Figure 3(b)).

### 3.5. Complete Freund Adjuvant-Induced Pain

**Figure 4:**
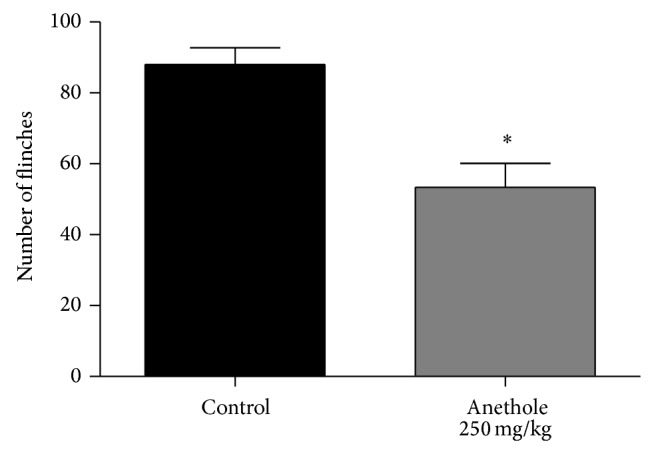
Effect of oral treatment of anethole (250 mg/kg) on number of flinches induced by 20 *μ*L of complete Freund adjuvant (CFA). Data are represented as mean ± S.E.M. ^*^ *P* < 0.05, compared with the control group (one-way ANOVA followed by Tukey test).

Complete Freund adjuvant induced an average of 88 ± 4.7 flinches in the control group 20 min after CFA injection. Anethole at a dose of 250 mg/kg reduced 39% the number of flinches induced by CFA (Figure 4).

### 3.6. Open-Field Test

**Figure 5:**
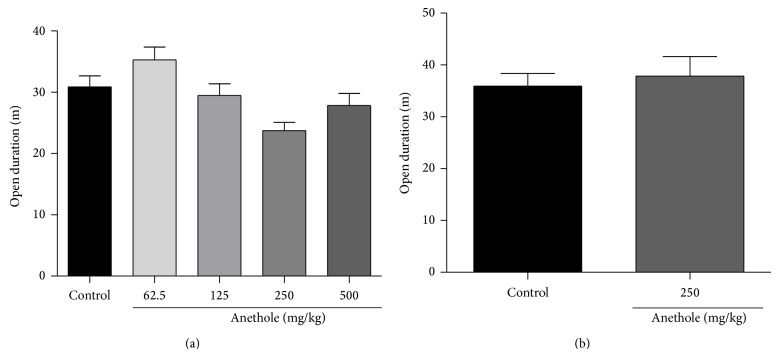
Effect of oral treatment of anethole on open-field test. The animals were treated with a single dose (62.5, 125, 250, and 500 mg/kg) and for 7 days (250 mg/kg) of anethole. Data are represented as mean ± S.E.M. ^*^ *P* < 0.05, compared with the control group (one-way ANOVA followed by Tukey test).

A major concern in experiments that evaluate the analgesic action of novel agents is whether pharmacological treatment causes other behavioral alterations, such as alterations in motor coordination or sedation that can be misinterpreted as analgesia. Treatment with different doses of anethole did not reduce duration locomotion in the open-field test compared with the control group (Figure 5(a)). After 7 days of daily treatment with anethole (250 mg/kg), the animals did not exhibit locomotor changes in the open-field test (Figure 5(b)).

## 4. Discussion

In this study, we demonstrated the antinociceptive effect of anethole in various experimental models of pain. Anethole significantly reduced peripheral nociception (i) in the abdominal constriction model, (ii) in the second phase of the formalin test, (iii) induced pain by CFA, and (iv) by glutamate. However, it had no central antinociceptive effect, with no effects in the hot-plate test or first phase of the formalin test.

The test of abdominal constriction induced by acetic acid has been used as a screening tool for the evaluation of anti-inflammatory and analgesic agents that act at the peripheral level. Acetic acid indirectly acts on the release of endogenous substances, such as glutamate, bradykinin, serotonin, histamine, and sympathomimetic amines, culminating in nociceptor activation. It also increases the release of prostaglandins that are responsible for nociceptor sensitization [13, 14]. Nociceptors that respond to acetic acid depend on the release of some cytokines, such as TNF, IL-1, and IL-8, from macrophages and mastocytes in the peritoneal cavity [15–17]. We verified that anethole reduced acetic acid-induced abdominal constrictions similar to indomethacin at doses of 250 and 500 mg/kg, indicating that the antinociceptive activity reached the maximum effect in these doses. This action may be attributable to the inhibition of one or more inflammatory mediators that are involved in this activity.

Given that the formalin test presents distinct phases, it is considered a useful tool for elucidating the mechanism of action of compounds because it allows differentiation between neurogenic/central pain (first phase) and inflammatory/peripheral pain (second phase). The first phase occurs immediately after an intraplantar injection of formalin and is characterized by intense neurogenic pain that is generated by the direct activation of nociceptors through C-fiber stimulation. The second and longer phase appears to be caused by the release of nociceptive mediators, such as histamine, serotonin, prostaglandin, and bradykinin [16, 18]. Analgesic drugs, such as narcotics, act via different mechanisms mainly at the central level in the initial and late phases of the test, inhibiting both phases equally. Drugs with peripheral action, such as dexamethasone and nonsteroidal antiinflammatory drugs, only inhibit the second phase of formalin-induced nociception [19, 20]. Pretreatment with anethole at different doses did not affect the nociceptive response in the initial phase (neurogenic pain) showing that it has no central effect; however, it significantly inhibited the number of flinches in the second phase of the test, indicating that the compound diminished peripheral pain.

To confirm this observation, we used the hot-plate test because it measures supraspinal (central) analgesia produced by drugs. Anti-inflammatory drugs with a peripheral mechanism of action have no effect on thermal nociception [21, 22]. Pretreatment with anethole at different doses did not increase the time spent on the hot-plate, indicating that it has no central antinociceptive action as also observed by formalin-induced nociception test.

Glutamate is the principal excitatory neurotransmitter in the central nervous system. Recently, the presence of glutamate receptors (GluRs) in peripheral sensorial terminal areas has also been reported, demonstrating its role in peripheral nociceptive transduction. Carlton [23] reported that an intraplantar injection of glutamate triggered pain-related behavior, suggesting that manipulation of peripheral glutamatergic systems can provide a new approach for the treatment of pain of peripheral origin. If so, then this may meet currently unmet clinical needs.

Experimentally, glutamate injection in the animals paws results in an intense, short-duration nociceptive response associated with the formation of paw edema. One of the mediators that are responsible for these events is nitric oxide. The release of nitric oxide increases the synthesis or release of other inflammatory mediators, such as cytokines and prostanoids, resulting in an increase in inflammatory and nociceptive responses [24]. Beirith et al. [12] demonstrated that nitric oxide inhibition reduces nociception and paw edema. Anethole may be a potential alternative therapeutic for the control of peripheral pain, given that it reduced nociception in several experimental models, including a test with glutamate. In the present study, we found that anethole treatment reduced both inflammatory and nociceptive responses. Thus, anethole may influence the synthesis or release of nitric oxide. This hypothesis is supported by the results of Domiciano et al. [7] that reported a reduction of nitric oxide levels in pleural exudate of animals treated with anethole.

The pain induced by CFA causes the activation or release of various endogenous inflammatory mediators, such as histamine, serotonin, and kinins, through the degranulation of resident mastocytes, in addition to an increase in prostaglandins caused by the activation of cyclooxygenases and cytokines, such as IL-1*β* and TNF, that stimulate nociceptors [16, 18, 24–26]. Anethole has been recently shown to reduce the synthesis or release of cytokines (IL-1*β* and TNF) and prostaglandin E_2_ in models of inflammation induced by CFA and carrageenan [7, 8]. Indeed, Ponte et al. [27] reported that anethole reduced paw edema produced by histamine, bradykinin, and serotonin, demonstrating the influence of anethole on these inflammatory mediators release. Our data showed that anethole treatment (250 mg/kg) in a single dose reduced the number of flinches induced by CFA demonstrating its antinociceptive activity probably by acting in mechanisms of different inflammatory mediators released during inflammatory process.

In this study, we also found that treatment with anethole in a single dose or daily administrated for 7 days did not alter motor activity in the open field test. Thus, our data indicates that anethole had no sedative effects at the doses tested. Importantly, when a compound has a sedative effect, this can interfere with the results in tests of nociception [28, 29].

Taken together, the data show that although the anethole has an important antinociceptive effect, the effective dose and the intensity of the effect are variable, according to the experimental model used, which may be dependent on the type and concentration of the mediators produced or released in the response.

## 5. Conclusions

The present work provides evidence that anethole exerts a peripheral antinociceptive effect without causing sedation. This antinociceptive action may be the result of a reduction of the production or release of inflammatory mediators. For all this we propose that the anethole might represent an interesting therapeutic alternative in inflammatory and painful diseases.

## Figures and Tables

**Table 1:** Reaction time of animals in hot plate test: 0, 15, 30, 60, and 90 min after treatment of anethole (62.5, 125, 250, and 500 mg/kg, p.o.) and meperidine (50 mg/kg, i.p.).

Treatment (mg/kg)	Reaction time (s)				
	0 min	15 min	30 min	60 min	90 min
Control	8.9 ± 0.5	8.4 ± 0.6	8.6 ± 0.5	7.9 ± 0.4	7.7 ± 0.9
Anethole 62.5	6.8 ± 0.5	6.8 ± 0.6	7.2 ± 1.1	10.7 ± 1.4	8.4 ± 0.6
Anethole 125	8.2 ± 0.4	8.5 ± 0.9	11.3 ± 1.1	9.1 ± 0.5	10.8 ± 1.0
Anethole 250	7.6 ± 0.7	11.9 ± 0.6	12.3 ± 0.8	11.1 ± 1.2	8.7 ± 0.5
Anethole 500	7.3 ± 0.4	10.9 ± 0.8	10.9 ± 1.2	10.5 ± 0.6	9.8 ± 0.7
Meperidine	7.6 ± 0.5	25.4 ± 1.7^*^	14.0 ± 1.3^*^	14.2 ± 1.2^*^	12.4 ± 0.7

Control: animals that received oral treatment of saline (10 mg/kg): Anethole 62.5, 125, 250, and 500: animals that received oral treatment of anethole in doses of 62.5, 125, 250, and 500 mg/kg, respectively; meperidine: animals that received intraperitoneal injection of meperidine in doses of 50 mg/kg. Data are represented as mean ± S.E.M. ^*^ *P* < 0.05, compared with the control group (one-way ANOVA followed by Tukey test).
